# Advantages of the dual-channel multi-bending endoscope for ERCP in patients with Billroth II reconstruction

**DOI:** 10.1055/a-2155-5608

**Published:** 2023-09-15

**Authors:** Haruka Toyonaga, Tsuyoshi Hayashi, Masayo Motoya, Toshifumi Kin, Kuniyuki Takahashi, Akio Katanuma

**Affiliations:** Center for Gastroenterology, Teine Keijinkai Hospital, Hokkaido, Japan

Endoscopic retrograde cholangiopancreatography (ERCP) is challenging in patients with surgically altered anatomy; in these cases, balloon-enteroscope-assisted ERCP can be performed 1 2 3 . In patients who have undergone distal gastrectomy with Billroth II reconstruction, the duodenal papilla is within the range of a normal endoscope. The dual-channel multi-bending scope (M-scope – GIF-2TQ260M; Olympus Corp., Tokyo, Japan) ( Fig. 1 ) has reported efficacy for ERCP in these patients 4 ; however, precise techniques and methodologies are lacking. Herein, we describe the advantages of using the M-scope in patients with Billroth II anatomy ( Video 1 ).

**Fig. 1:**
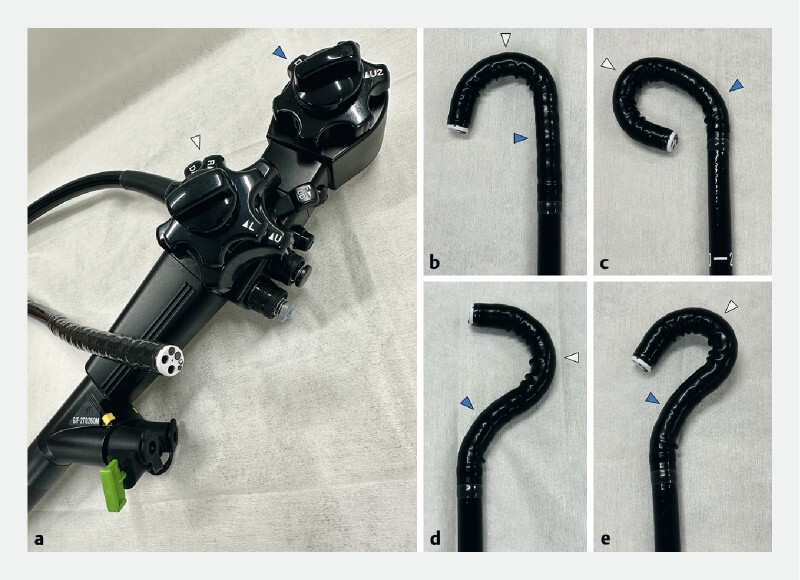
The M-scope (GIF-2TQ260M; Olympus Corp., Tokyo, Japan). **a** The scope has dual working channels and two bending sites (Scope tip diameter φ11.7 mm, channel diameter φ3.2 mm/φ3.2 mm). The two bending sites allow for a variety of scope positions: **b** first angle up and second angle neutral; **c** first angle up and second angle up; **d** first angle up and second angle down; **e** first angle fully up and second angle down. White arrow, first bending site; blue arrow, second bending site.

**Video 1** Advantages of a dual-channel multi-bending endoscope for endoscopic retrograde cholangiopancreatography in patients with Billroth II reconstruction.


The M-scope can overcome even acute angles by bending in two places sequentially ( Fig. 2 ). On reaching the papilla, maintaining a frontal view is difficult because of the tangential scope position and the scope tip becoming embedded in the mucosa. Altering the second angle moves the scope away from the papilla allowing a frontal view without mucosal embedding ( Fig. 3 ).

**Fig. 2:**
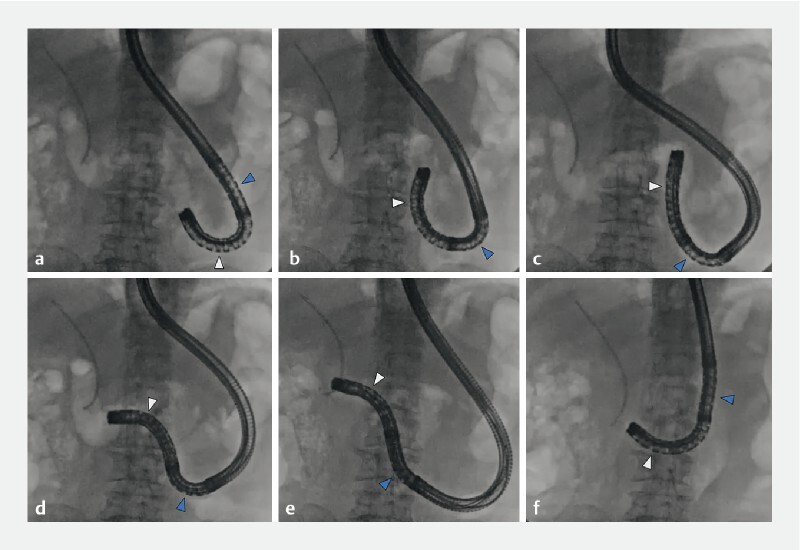
The endoscope can negotiate the acute angles encountered in patients with surgically altered anatomy. **a** Moving only the first angle upward cannot overcome the acute angle, and leads only to extension of the intestine. **b,c** By adjusting the second angle upwards, the acute angle was easily negotiated and the scope tip was guided more deeply. **d** In order to round the next bend, the first bending site was angled downwards to capture the lumen. **e** The second bending site was set to neutral, enabling deeper insertion of the scope tip. **f** The short position of the scope allows stability. White arrow, first bending site; blue arrow, second bending site.

**Fig. 3:**
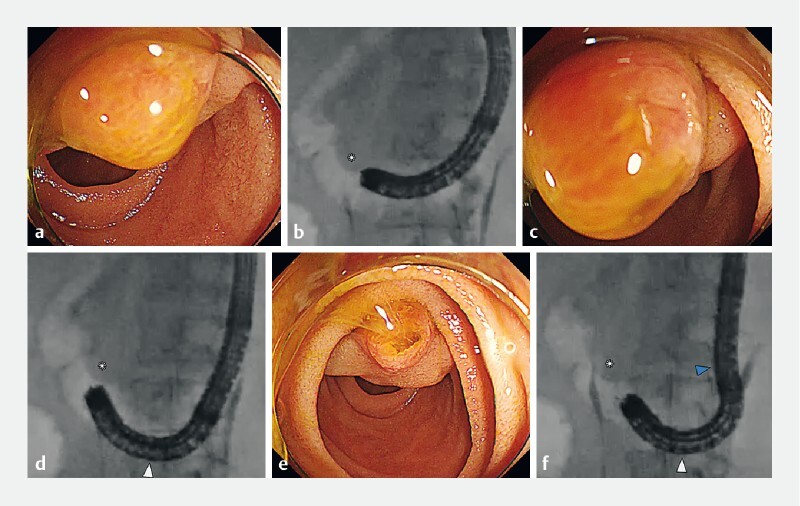
Endoscopic and fluoroscopic views. **a, b** In the short scope position, the endoscope and the papilla are tangential, and the biliary orifice is not visible. **c, d** During maneuvering of the first bending site, the endoscope tip fails to maintain a sufficient distance from the papilla and becomes embedded in the mucosa; the field of view cannot be secured. **e, f** By manipulating the second bending site in combination with the first, it is possible to obtain an appropriate distance while maintaining a frontal view of the papilla. Asterisk, papilla; white arrow, first bending site; blue arrow, second bending site.

Biliary cannulation can be challenging in patients with Billroth II anatomy because of high papillary mobility or its angle relative to the scope. The dual channel of the M-scope facilitates simultaneous holding and pulling of the papilla and biliary cannulation ( Fig. 4 ). This system also allows separate access for devices, such as the needle-knife and guidewire during sphincterotomy, preventing interference.

**Fig. 4:**
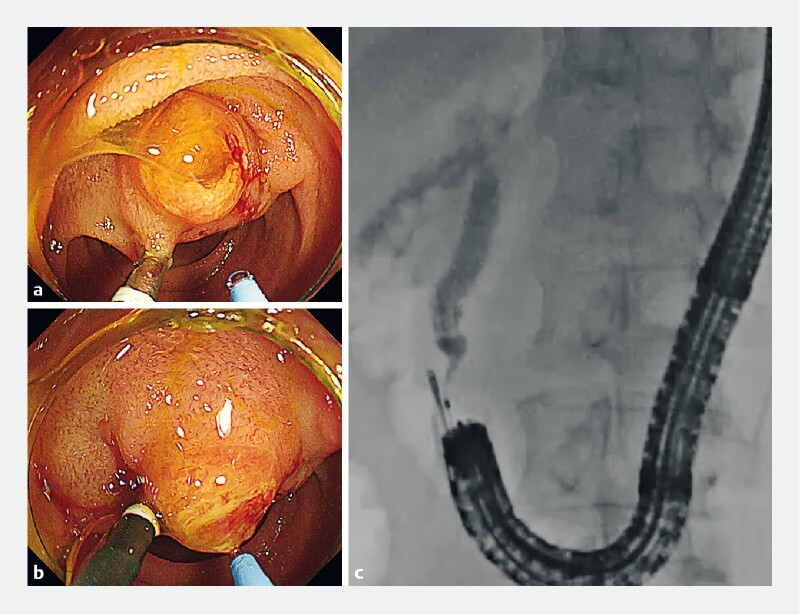
Use of the scope during cannulation. **a–c** Using the two channels of the endoscope, forceps inserted through one channel can be used to hold and pull the papilla during biliary cannulation, and a catheter can be inserted through the other channel.

Performing sphincterotomy is often difficult in cases of altered anatomy because scope position adjustments are not straightforward. These adjustments are made easier by utilizing both bending sites ( Fig. 5 a–c ). Furthermore, when performing endoscopic papillary large balloon dilation, the position of the balloon and scope must be fine-tuned to prevent the balloon from slipping; the multi-bending function allows the balloon position to be adjusted without scope position adjustments ( Fig. 5 d–f ).

**Fig. 5:**
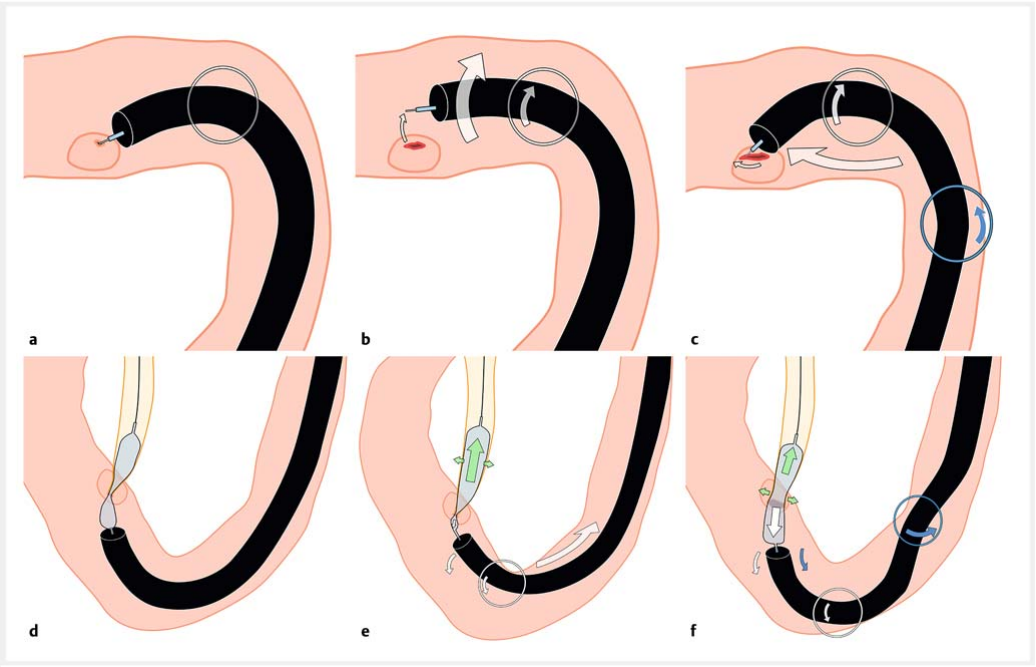
Illustration depicting a needle-knife sphincterotomy and endoscopic papillary large balloon dilation (EPLBD) using the multi-bending maneuver. **a, b** Using only the first bending position, the tip of the knife deviates from the submucosa with each incision. In addition, moving the needle tip with the push/pull of the scope may produce unpredictable results, particularly in patients with surgically altered anatomy. **c** By utilizing both the first and second bending sites, an appropriate angle can be created without moving the scope push/pull, thereby allowing for an efficient and safe incision. **d, e** Inflation of the EPLBD balloon creates a force that pulls the balloon into the bile duct (green arrow). To adjust to the proper position, the scope tip is moved away by pulling the scope and manipulating the angle of the first bend. However, it is difficult to exert sufficient force to pull the balloon, making fine adjustments difficult. **f** The second bending site can be used in conjunction with the first to fine-tune the distance of the scope tip without pulling the scope, thereby allowing the balloon position to be adjusted while maintaining a stable scope position. White circle, first bending site; blue circle, second bending site.

If these features were incorporated into the balloon endoscope, it could further facilitate ERCP in cases with altered surgical anatomy.

Endoscopy_UCTN_Code_TTT_1AR_2AK

## Notes

Endoscopy E-Videos

https://eref.thieme.de/e-videos
*E-Videos* is an open access online section of the journal *Endoscopy* , reporting on interesting cases and new techniques in gastroenterological endoscopy. All papers include a high-quality video and are published with a Creative Commons CC-BY license. Endoscopy E-Videos qualify for HINARI discounts and waivers and eligibility is automatically checked during the submission process. We grant 100% waivers to articles whose corresponding authors are based in Group A countries and 50% waivers to those who are based in Group B countries as classified by Research4Life (see: https://www.research4life.org/access/eligibility/ ). This section has its own submission website at https://mc.manuscriptcentral.com/e-videos
